# Sensory perception testing by monofilaments in the digits of controls and workers with HAVS

**DOI:** 10.1007/s00420-020-01523-8

**Published:** 2020-02-20

**Authors:** C. J. M. Poole, E. W. Robinson, G. Frost

**Affiliations:** 1 Centre for Workplace Health, Health and Safety Executive, Harpur Hill, Buxton, SK17 9JN UK; 2 Analysis and Data Group, Health and Safety Executive, Harpur Hill, Buxton, SK17 9JN UK

**Keywords:** Hand-arm vibration syndrome, HAVS, Monofilaments, Sensory perception, Neuropathy

## Abstract

**Objective:**

To determine if heavy manual work affects sensory perception in the digits and whether Semmes–Weinstein monofilaments (SWM) can be used as a screening tool to detect sensory neuropathy in the digits of workers exposed to hand-transmitted vibration (HTV).

**Methods:**

A cross-sectional study of office workers, heavy manual workers not exposed to HTV and workers with hand-arm vibration syndrome (HAVS). Sensory perception was measured in the digits by SWM using a forced-choice method to determine variability by sex, age, hand and digit. Frequency distributions were used to determine limit values and linear weighted kappa for intra-digit variability. Poisson regression was used to explore the relationship between sensory perception by SWM and abnormalities of thermal and vibration perception in the hands of workers with HAVS.

**Results:**

The sensory perception threshold of office workers did not vary by hand or digit. It was significantly lower in women < 30 than women aged ≥ 30 years. The 95th percentile for heavy manual workers was 1.00 (95% CI 0.60–1.00) and significantly higher than for office workers at 0.16 (95% CI 0.16–0.16). Heavy manual workers > 50 years had the highest threshold at 1.40 (95% CI 1.00–2.00). Weighted kappa for reliability was 0.63 (95% CI 0.53–0.70). A mean SWM threshold of ≥ 1.0 gram-force had a 79% sensitivity and 64% specificity for detecting abnormalities of thermal and vibration perception in the ipsilateral index and little fingers of workers with HAVS.

**Conclusions:**

SWM are a useful screening tool for detecting sensory loss in the digits of workers exposed to HTV.

## Introduction

The diagnosis of sensory neuropathy in the digits of workers exposed to hand-transmitted vibration (HTV) is challenging. The use of Semmes–Weinstein monofilaments (SWM) has been recommended by the Health and Safety Executive (HSE) in Guidance L140 on hand-arm vibration (HSE 2005) and in the latest classification of hand-arm vibration syndrome (HAVS) (Poole et al. 2019). SWM may also be used by diabetologists and hand surgeons. Their use has been endorsed by the American Peripheral Neuropathy Association because of their utility in clinical settings (PNA 1993), but their validity in determining sensory neuropathy in workers with HAVS is uncertain. Quantitative sensory perception tests (QST) of thermal and vibration perception are also used to do this, but they are time consuming and only available in a few specialised centres.

The SWM method relies on the principle that a nylon filament will buckle when compressed according to its length, diameter, and the type of material used to make it. The force of application is then limited by the buckling load. Once buckled, the force imparted by the filament should be constant.

Monofilaments work by stimulating light-touch and mechanoreceptors in the epidermis and dermis of the skin. Stimulation causes ion exchange in the receptor, which then sends an action potential along myelinated (A fibre) and non-myelinated (C fibre) afferent nerves to the dorsal ganglia of the spinal cord and then up to the somatosensory cortex of the brain (Guyton and Hall 2016). Some monofilaments are ‘soft tipped’ to avoid stimulating nociceptors in the skin and force overshoot. A full set of SWMs has 20 monofilaments with bend forces that range from 0.008 to 300 gram-force (g-f), but hand and foot sets can be purchased with five filaments in a set.

Unfortunately, their method of use has not been standardised and some methods are better suited to a laboratory than a medical clinic. Methods that have been used include forced-choice staircase algorithms with filaments applied in ascending, descending or random order, sometimes with additional auditory cues and ‘catch’ trials. The threshold can be taken as the lowest force felt, or the mean between the lowest felt and the next lowest monofilament, or the mean of a series of applications (PNA 1993; Berquin et al. 2010; Tracey et al. 2012). Testing relies on the co-operation of the subject and may therefore be described as a psychophysical test.

Monofilaments have been shown to vary in performance by make (Booth and Young 2000; Lavery et al. 2012) and to decrease in bend force with an increase in ambient temperature or humidity (Werner et al. 2011; Haloua et al. 2011) as well as with repeated loading (Booth and Young 2000; Lavery et al. 2012). In one study, tolerance (± 10%) of a calibrated 10 g-f monofilament was found to vary with the number of times the filament was buckled, with only 80% of monofilaments remaining in the tolerance range after 100 compressions (Booth and Young 2000).

The sensory perception threshold of the digits in normal healthy subjects has been shown to increase with age (Thornbury and Mistretta 1981; Schulz et al. 1998), but the effect of sex or handedness is less clear cut (Thornbury and Mistretta 1981; Schulz et al. 1998; Collins et al. 2010). In one study, mean sensory perception by digit in men over age 55 ranged between 0.27 (little finger) to 0.40 (thumb) g-f (Schulz et al. 1998). The epidermis of the skin is known to thicken and harden with heavy manual work, so it would be reasonable to expect the sensory perception threshold to rise in such workers, particularly if gloves are not worn. There is one small study which showed heavy and moderate work to be associated with a significantly higher SWM threshold than light work (Birke et al. 2000).

Our study was undertaken to ascertain normal sensory perception by sex, age, hand and digit using SWM in office workers and the effect of heavy manual work on sensory perception. The results were compared with a group of workers diagnosed with neurological HAVS. The relationship between SWM and QST in workers with HAVS was also investigated.

## Methods

### Study population

Sensory perception was determined on the pulps of all the digits of both hands in three study populations: (1) office workers; (2) heavy manual workers not exposed to HTV and (3) workers with HAVS. The office workers were all employees of the HSE, who spent most of their time using computers, were tested by the same investigator. Any office worker who undertook heavy manual work, played competitive sport with their hands, or had a medical problem that could affect sensory perception in the hands, such as carpal tunnel syndrome, was excluded from the study. All subjects were tested whilst comfortably seated in a quiet room.

The heavy manual workers were bricklayers, roofers and scaffolders who were working on large commercial building sites in the UK. Those who had regularly used vibrating tools, such as labourers, were excluded. They were all tested by the same investigator. The population with HAVS was made up of workers who were judged by the referring occupational physician to have been exposed to enough HTV to get HAVS. They had been referred to the HSE’s Science and Research Centre, Buxton, UK for high level (Tier 5) HAVS health surveillance with quantitative sensory tests of the digits. All of them were examined by CJMP and as appropriate confirmed to have neurological HAVS. They were then staged according to the International Consensus Criteria (ICC) (Poole et al. 2019), which unlike the Stockholm Workshop Scale, is prescriptive in how sensory perception loss should be determined. No cases of neurological HAVS were excluded during the collection period unless they were associated with median or ulnar nerve neuropathy.

There were no available data to indicate the likely size of the difference in SWM thresholds between the three study populations. A large sample of office workers was recruited to define demographic parameters such as age and sex, however the size of the two smaller groups (heavy manual workers and HAVS cases) was determined by the numbers required to set limit values and the constraints of the budget.

### Sensory perception testing

Sensory perception thresholds were determined using a full set of SWM in handsets of five consecutive filaments (0.04–0.6 g-f; 1.0–6.0 g-f; 8-60 g-f) supplied by Connecticut Bioinstruments, New York, USA. Bend forces were guaranteed by the manufacturer within 15% tolerance. The filaments were checked at three monthly intervals by the investigators to make sure they were still in tolerance by way of a jig and Mitutoyo height gauge, which applied each filament at right angles to a pressure plate of a fast-responding Precisa digital balance. After a short period of familiarisation of the technique with the subject, filaments were gently applied perpendicular to the surface of the pulp of each digit in sequence, avoiding any obvious callosities, from little finger to thumb, by the investigator until they buckled. The subject was asked to close their eyes during testing. No more than 10 subjects were tested per day. The filament was held in contact with the digit for about a second. They were applied in ascending order of bend force using the ‘two out of three’ method. That is, the smallest filament to be felt twice out of a maximum of three applications was taken as the sensory threshold for the digit being tested. The time to test all digits in one hand is about 5 min and for this reason this method was thought to be the most practical for busy practitioners in a clinic.

### Analysis and statistics

For each study population, the ‘normal’ cut-off was estimated using the 95th percentile of the sensory thresholds of each digit. As the bend forces chosen for this study are not truly continuous, it would not be appropriate to assume a particular distribution so the 95th percentile was taken directly from the data. The 95% confidence intervals (95% CI) were bootstrapped with the subject as the resampling unit, thereby taking into account any clustering due to multiple digits per person. Results were analysed by sex, age, hand and digit for each study population. Because of the method used to estimate the 95th percentiles and CIs, no formal statistical tests were undertaken to compare the 95th percentiles across groups. Instead it was assumed that if the CIs did not overlap, then the two values were significantly different at the *p* < 0.05 level. The *t* test was used to compare group ages.

To determine intra-digit variability, 20 office workers and 10 heavy manual workers were re-tested in the same way and by the same investigator two to four weeks later. The degree of agreement between the two measurement occasions was assessed using the percentage agreement and linear weighted kappa. The 95% CIs were bootstrapped with the subject as the resampling unit.

For workers with HAVS, the mean sensory perception threshold of the two digits with the highest thresholds was ascertained. This was compared with the number of abnormal thermal (hot and cold) and vibration perception (31.5 and 125 Hz) thresholds in the index and little fingers of the same hand. On the same day, thermal aesthesiometry was undertaken according to the method described by Lindsell and Griffin 2003 and vibration perception according to ISO 13091–2 (2003) and as described (Poole et al. 2016). Thresholds > 48.5 °C for hot or < 19.0 °C for cold; > 0.4 m/s^2^ for 31.5 Hz or > 1.0 m/s^2^ for 125 Hz were taken as abnormal (HSE 2005; Lindsell and Griffin 2003), making the maximum QST score per finger four.

The relationship between the mean SWM threshold of the two digits with the highest thresholds and the number of QST abnormalities in the same hand of subjects with HAVS was modelled using mixed effects Poisson regression with the subject as the random effect and using a restricted cubic spline with three knots. The small number of results ≥ 10 g-f were excluded. The utility of SWM for identifying abnormalities of QST was assessed using Receiver Operating Characteristic (ROC) curves and sensitivity and specificity calculated at different sensory thresholds.

## Results

There were 300 office workers of whom 155 were male, 144 female and one unstated. Their mean age was 42 (range 19–68) years. There were 272 who declared their right-hand and 27 their left-hand to be dominant, with one declaring ambidexterity. The ambient temperature of the laboratory varied between 22–24 °C and humidity between 30 and 35%. Table 1 shows sensory perception broken down by sex, age, digit and hand-dominance. The 95th percentile was 0.16 g-f and did not vary by sex, hand-dominance or digit. Women < 30 had a significantly lower 95th percentile for sensory perception than women ≥ 30 years. Men had a higher 95th percentile for the dominant thumb (0.40 g-f) compared with the other digits, but as the CIs overlapped the difference did not reach statistical significance.

**Table 1:** 95th percentiles of sensory perception thresholds for the digits of men and women office workers

	Men				Women			
	Number of subjects	Number of digits	95th percentile	95% confidence interval^a^	Number of subjects	Number of digits	95th percentile	95% confidence interval^a^
All	155	1549	0.16	(0.16–0.16)	144	1440	0.16	(0.16–0.16)
Age								
< 30 years	18	180	0.16	(0.16–0.16)	37	370	0.07	(0.07–0.07)
30–39 years	32	320	0.16	(0.16–0.16)	32	320	0.16	(0.12–0.16)
40–49 years	54	540	0.16	(0.16–0.16)	37	370	0.16	(0.16–0.16)
50 + years	51	509	0.16	(0.16–0.40)	38	380	0.16	(0.16–0.16)
Digit								
Little	155	309	0.16	(0.16–0.16)	144	288	0.07	(0.07–0.16)
Ring	155	310	0.16	(0.16–0.16)	144	288	0.07	(0.07–0.16)
Middle	155	310	0.16	(0.16–0.16)	144	288	0.16	(0.07–0.16)
Index	155	310	0.16	(0.16–0.16)	144	288	0.16	(0.16–0.16)
Thumb	155	310	0.16	(0.16–0.40)	144	288	0.16	(0.16–0.16)
Hand dominance^b^								
Dominant	154	769	0.16	(0.16–0.16)	144	720	0.16	(0.16–0.16)
Non-dominant	154	770	0.16	(0.16–0.16)	144	720	0.16	(0.12–0.16)
Hand dominance and digit^b^								
Dominant Little	153	153	0.16	(0.16–0.16)	144	144	0.16	(0.07–0.16)
Dominant Ring	154	154	0.16	(0.16–0.16)	144	144	0.07	(0.07–0.16)
Dominant Middle	154	154	0.16	(0.16–0.16)	144	144	0.16	(0.07–0.16)
Dominant Index	154	154	0.16	(0.16–0.16)	144	144	0.16	(0.16–0.16)
Dominant Thumb	154	154	0.40	(0.16–0.40)	144	144	0.16	(0.16–0.16)
Non-dominant Little	154	154	0.16	(0.16–0.16)	144	144	0.07	(0.07–0.16)
Non-dominant Ring	154	154	0.16	(0.16–0.16)	144	144	0.07	(0.07–0.16)
Non-dominant Middle	154	154	0.16	(0.16–0.16)	144	144	0.07	(0.07–0.16)
Non-dominant Index	154	154	0.16	(0.16–0.16)	144	144	0.16	(0.07–0.16)
Non-dominant Thumb	154	154	0.16	(0.16–0.40)	144	144	0.16	(0.16–0.16)

^a^Bootstrapped 95% percentile confidence interval

^b^One male was ambidextrous so their results (10 digits) were excluded when investigating hand dominance

The frequency distribution of sensory perception for the office workers showed the median to be 0.07 g-f with no digit with a threshold greater than 0.6 g-f (Table 2). The data were positively skewed towards the lower sensory thresholds.

**Table 2:** Frequency distribution of sensory perception thresholds for the digits of office workers and heavy manual workers

Threshold (g–f)	Office workers (*n* = 300)			Heavy manual workers (*n* = 115)		
	Frequency	Percent	Cumulative percent	Frequency	Percent	Cumulative percent
0.04	1144	38.1	38.1	90	7.8	7.8
0.07	1359	45.3	83.5	239	20.8	28.7
0.16	462	15.4	98.9	467	40.7	69.4
0.40	27	0.9	99.8	184	16.0	85.4
0.60	7	0.2	100.0	102	8.9	94.3
1.0	0	0.0	100.0	44	3.8	98.2
1.4	0	0.0	100.0	14	1.2	99.4
2.0	0	0.0	100.0	7	0.6	100.0
Total	2999^a^	100.0		1147^b^	100.0	

^a^One person missing result for one finger

^b^Two people missing three results in total

There were 115 heavy manual workers all of whom were male. Their mean age was 40 (range 18–66) years, which was not significantly different from the office workers (*p* = 0.114). There were 99 who declared their right-hand and 14 their left-hand to be dominant, with one declaring ambidexterity and one unstated. There was no difference in sensory perception by hand dominance. The 95th percentile for the dominant thumb was 1.00 g-f (95% CI 1.00–1.40), which was significantly higher than the middle 0.60 g-f (95% CI 0.60–0.60) and the ring fingers 0.60 g-f (95% CI 0.40–0.60) on the dominant hand.

Table 2 shows the frequency distribution of the sensory perception thresholds for the office and heavy manual workers. The median threshold for heavy manual workers was 0.16 g-f and there was no threshold > 2.00 g-f. The 95th percentile was 1.00 g-f (95% CI 0.60–1.00), which was significantly greater than for office workers 0.16 g-f (95% CI 0.16–0.16). Heavy manual workers ≥ 50 years had the highest 95th percentile of 1.4 g-f (95% CI 1.00–2.00), but there was no consistent trend with age; 40–49 years 0.60 g-f (95% CI 0.40–1.00); 30–39 years 0.40 g-f (95% CI 0.28–0.60); < 30 years 0.60 g-f (95% CI 0.60–1.00).

Table 3 shows the degree of agreement between repeated measurements on the same digits. There was perfect agreement for 198/300 (66%) of digits; 92/300 (31%) differed by one filament; 10/300 (3%) differed by two filaments. No finger differed by more than two filaments. The weighted kappa statistic for intra-subject reliability was 0.63 (95% CI 0.53–0.70). The degree of agreement for the office workers was not substantially different from that of the heavy manual workers.

**Table 3:**
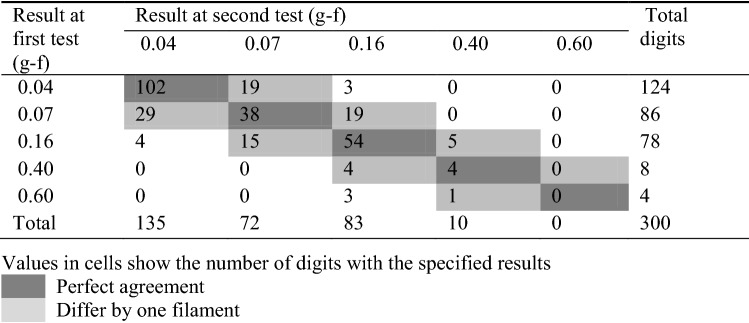
Agreement between the first and second tests for identical digits of office workers and heavy manual workers

There were 62 cases of neurological HAVS all of whom were male. Their mean age was 51 (23–69) years. They were older than the office (*p* < 0.001) and the heavy manual workers (*p* < 0.001). There were 55 who declared their right-hand and 7 their left-hand to be dominant. There were no significant differences in the 95th percentiles of sensory perception by age, digit or hand dominance. Of the 124 hands that were classified and neurologically staged according to the ICC (Poole et al. 2019); 4 were stage 0; 66 were stage 1; 36 were stage 2 and 18 were stage 3.

For workers with HAVS the median threshold of perception by SWM was 0.6 g-f and 11 digits had a threshold ≥ 10 g-f. The 95th percentile was 4.0 g-f (95% CI 2.0–6.0), which was significantly greater than the office workers and the heavy manual workers (confidence intervals not overlapping). Table 4 shows the frequency distribution of sensory perception thresholds for workers with HAVS.

**Table 4:** Frequency distribution of sensory perception thresholds for the digits of workers with HAVS

Threshold (g–f)	Frequency	Percent	Cumulative percent
0.04	4	0.6	0.6
0.07	41	6.6	7.3
0.16	175	28.3	35.5
0.4	89	14.4	49.9
0.6	80	12.9	62.8
1.0	95	15.3	78.2
1.4	52	8.4	86.6
2.0	34	5.5	92.1
4.0	27	4.4	96.4
6.0	11	1.8	98.2
> 10	11	1.8	100.0
Total	619^a^	100.0	

^a^One person missing one digit

**Fig. 1:**
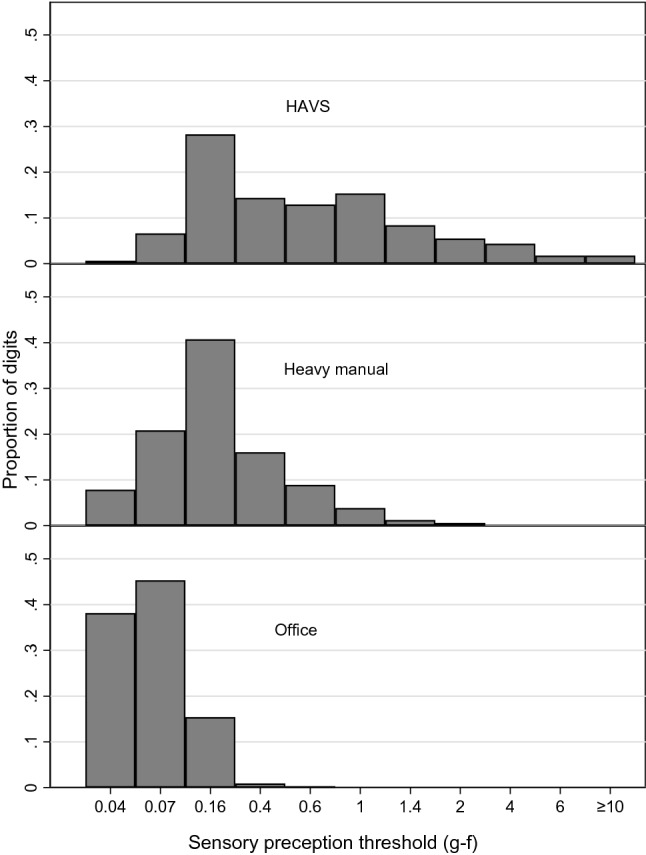
Semmes–Weinstein monofilament sensory perception thresholds of the digits of office workers, heavy manual workers and workers with HAVS

Figure 1 shows the spread of sensory perception in the digits by SWM for the three study populations. The range increased from office workers (0.04–0.60 g-f), to heavy manual workers (0.04–2.0 g-f), to HAVS cases (0.04 to ≥ 10 g-f).

**Fig. 2:**
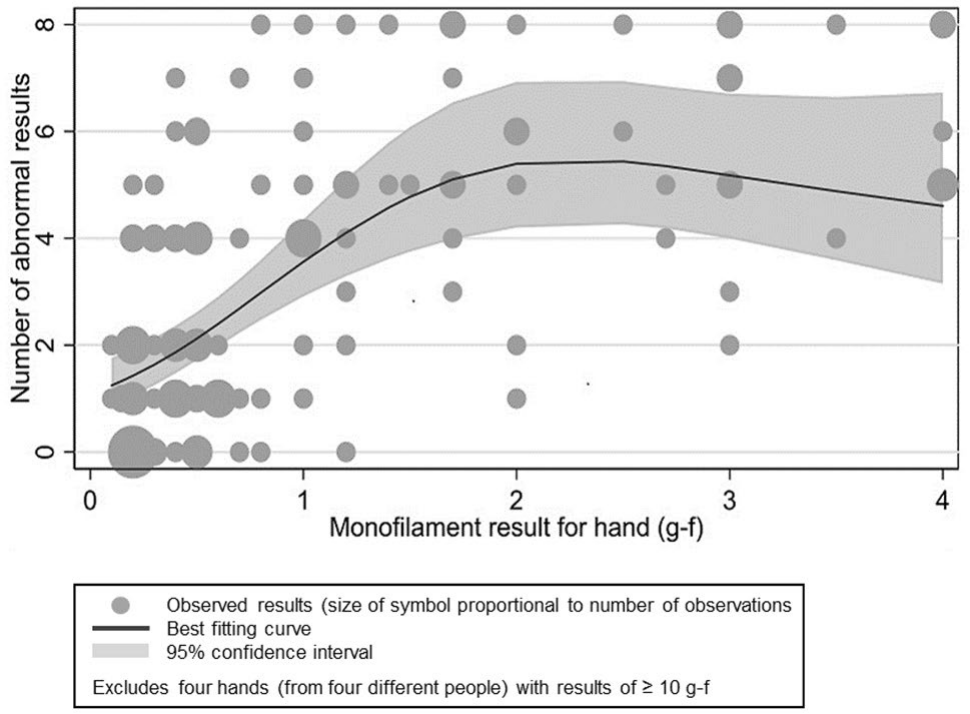
Best fit Poisson regression line between Semmes Weinstein monofilament thresholds and the number of abnormal thermal and vibration perception thresholds in the hands of workers with HAVS

The relationship between the SWM threshold and the total number of thermal and vibration threshold abnormalities in 120 hands of workers with HAVS is shown in Fig. 2. There was an increase in the number of abnormal thresholds as the SWM bend force increased, reaching a plateau at about 2 g-f. A fitted linear spline with a knot at 2 g-f indicated that, when the SWM bend force was < 2 g-f, the number of abnormal QST thresholds approximately doubled with each 1 g increase in force (95% CI 1.56–2.58). Similar curves were obtained when the SWM thresholds were compared separately with the number of abnormal thermal or vibration results.

The AUC for abnormalities of thermal and vibration perception in the index and little fingers in the same hand of workers with HAVS was 0.78 (95% CI 0.67–0.87) and for abnormalities of thermal or vibration perception was 0.84 (95% CI 0.74–0.90). For the former, sensitivity was 100% when the SWM bend force was 0.4 g-f, decreasing as the bend force increased. Specificity increased as the SWM bend force increased reaching 100% at ≥ 10 g-f. A SWM cut-off of ≥ 1.0 g-f gave a sensitivity of 79% and a specificity of 64% for abnormalities of thermal and vibration perception. The corresponding sensitivity and specificity for abnormalities of thermal or vibration perception was 68% and 89%, respectively. Table 5 shows sensitivities and specificities of SWM thresholds for detecting abnormalities of QST in workers with HAVS.

**Table 5:** Sensitivity and specificity of SWM thresholds for detecting abnormalities of thermal and/or vibration perception in the same hands of workers with HAVS

SWM threshold (g–f)^a^	Abnormalities of thermal and vibration perception in the hand		Abnormalities of thermal or vibration perception in the hand	
	Sensitivity (%)	Specificity (%)	Sensitivity (%)	Specificity (%)
≥ 0.15	100.00	2.11	100.00	4.26
≥ 0.2	100.00	4.21	100.00	8.51
≥ 0.3	100.00	23.16	94.81	38.30
≥ 0.4	100.00	30.53	89.61	44.68
≥ 0.5	93.10	41.05	81.82	57.45
≥ 0.6	89.66	53.68	74.03	72.34
≥ 0.7	89.66	58.95	72.73	80.85
≥ 0.8	86.21	62.11	70.13	85.11
≥ 1.0	79.31	64.21	67.53	89.36
≥ 1.2	68.97	71.58	55.84	91.49
≥ 1.4	65.52	77.89	50.65	97.87
≥ 1.5	62.07	78.95	48.05	97.87
≥ 1.7	62.07	80.00	46.75	97.87
≥ 2.0	51.72	84.21	37.66	97.87
≥ 2.5	41.38	87.37	31.17	100.00
≥ 2.7	34.48	87.37	28.57	100.00
≥ 3.0	31.03	88.42	25.97	100.00
≥ 3.5	13.79	91.58	15.58	100.00
≥ 4.0	10.34	92.63	12.99	100.00
≥ 10	3.45	96.84	5.19	100.00

^a^Mean threshold of the two digits with the highest thresholds

## Discussion

The sensory perception thresholds, as measured by SWM, in the digits of heavy manual workers not exposed to HTV was found to be significantly higher than that of office workers. This is probably because of thickening or hardness of the skin, but sensory neuropathy from trauma to the hands cannot be excluded. To date clinicians have been advised to take 0.2 g-f as the cut-off from normal (Lawson 2018), but by so doing they may be misdiagnosing thick or hard skin as stage 2 neurological HAVS. From these data the cut-off from normal, or 95th percentile for male heavy manual workers should be 1.0 g-f, and for men ≥ 50 years 1.4 g-f. The latter threshold is in keeping with the 95th percentile of 2 g-f for heavy manual workers not exposed to HTV in Italy (Poole et al. 2019). Based on this, the regression line in Fig. 2 and the AUC analysis, the optimum time for practitioners to refer workers exposed to HTV to specialised centres for thermal and vibration sensory perception tests would appear to be when the mean SWM bend force in two digits of a hand, ideally not supplied by the same nerve, is ≥ 1.0 g-f. In this way, SWM can be used as a screening tool for the more sophisticated and expensive tests of thermal and vibration perception. Lowering the SWM threshold for referral would increase the sensitivity for identifying abnormalities of thermal and vibration perception but reduce its specificity. Lowering the threshold by one filament to ≥ 0.6 g-f would take into consideration the reliability of the method in that 97% of intra-subject results were identical or differed by one filament.

The overlap in sensory perception of some office workers and heavy manual workers may be because some of the heavy manual workers wore gloves, in which case their skin would be expected to be like that of an office worker. The absence of a significant difference in sensory perception by hand or digit indicates that these factors do not have to be taken into consideration when determining abnormality in workers with potential neuropathy. The increased sensitivity of the digits of women office workers < 30 years may have occurred by chance, but as this finding is biologically plausible it is likely to be a true finding.

The overlap in sensory perception thresholds of the heavy manual workers not exposed to HTV and workers with HAVS could be expected as some of the workers with HAVS had an early stage of HAVS with only neurological symptoms and some of the heavy manual workers would have had thick or hard skin or sub-clinically damaged hands. By comparison, reduced sensory perception has been reported in the feet where the highest SWM threshold was found over the heels and the lowest over the arches in keeping with the thickness and hardness of the skin (Strzalkowski et al. 2015). Recent work has shown quantitative sensory tests (QST) to be unaffected by the thickness of the skin in the digits (Lundstrom et al. 2018). Doctors have been recommended to use SWM (HSE 2005), but their validity has been uncertain because of the absence of normative data and their unknown relationship with the results of QST. These questions have now been answered. Furthermore, most clinicians in a community clinic setting will have access to SWM, but not to QST, so the optimum time to refer a worker for QST is important to know.

A weighted kappa statistic of 0.63 for repeated intra-digit testing with SWM indicates moderate to substantial reliability (Landis and Koch 1977). This was achieved when the same trained tester was used for each population. This is reassuring, but it should be noted that the technique for the use of SWM needs to be taught and practised. Higher reliability has been reported for the plantar surface of the great toe with coefficients > 0.9 with more complex testing algorithms lasting 20 min (Tracey et al. 2012). The method of application should be standardised, and the bend forces of the filaments validated at regular intervals. Fatigue and deterioration in bend force of the filaments with repeated use is less relevant in HAVS practice as normally only a few workers will be tested in any one day. Care needs to be taken to avoid stimulating nociceptors and the duration of each filament’s contact with the skin of a digit needs to be long enough to stimulate light touch and mechanoreceptors. Unless their use is well taught and practised, then the reliability of results between practitioners is likely to be poor. More sophisticated methods of application can be used in a laboratory setting, such as the method of limits, or multiple applications of the same force, or mechanical methods of application, but such accuracy is probably unnecessary when SWM are being used to screen workers for more accurate testing.

The strength of this work is that relatively large populations of workers have been studied in a standardised way with the same SWM by the same investigators. It is not known how a different technique with a more complex algorithm would affect results, but the method described is quick and easy to use in a busy clinic setting and suitable for screening. We used the mean of the two digits with the highest SWM thresholds and compared them with the QST results for the index and little fingers because this is how clinical testing is undertaken. We do not believe that comparing SWM and QST results of only the index and little fingers would have made an appreciable difference to our results. As with all psychophysical methods, conscious bias cannot be eliminated, but these data should help with its identification. For the office workers and the workers with HAVS, SWM testing took place in controlled ambient temperatures and humidity. Testing of the heavy manual workers took place at the workplace in portacabins where the ambient conditions could not be controlled, but we do not believe this made a substantial difference to our results. Based on this research, clinicians should be careful to purchase handsets of SWM that include filaments with bend forces in the range 0.2–2.0 g-f and not a standard WEST handset in the range 0.07–200 g-f.

## Conclusion

We have shown that heavy manual work increases the sensory perception thresholds of the digits in the hands as measured by SWM. The intra-subject reliability of SWM is good when a simple forced-choice method is used by the same assessor. In workers with HAVS, abnormalities of thermal and vibration perception increase as the SWM threshold increases up to a bend force of 2 g-f. We recommend that workers exposed to HTV have their digits screened with SWM and are referred for QST when the mean SWM bend force in two digits is ≥ 0.6 g-f.
